# Effectiveness of a school-based mental health intervention for school teachers in urban Pakistan: a randomized controlled trial

**DOI:** 10.1186/s13034-022-00470-1

**Published:** 2022-05-03

**Authors:** Nazish Imran, Atif Rahman, Nakhshab Chaudhry, Aftab Asif

**Affiliations:** 1 Department of Child and Family Psychiatry, King Edward Medical University/Mayo Hospital, Lahore, Pakistan; 2 Department of Primary Care and Mental Health, University of Liverpool, Liverpool, United Kingdom; 3 Department of Basic Sciences, King Edward Medical University/Mayo Hospital, Lahore, Pakistan; 4 Department of Psychiatry Behavioural Sciences, King Edward Medical University, Lahore, Pakistan

**Keywords:** Mental Health, Schools, Teachers, Intervention, Mental health literacy

## Abstract

**Background:**

Schools have a major role in promoting children’s physical and psychological health and well-being and the mental health literacy of all key stakeholders, especially teachers, is critical to achieving this goal. Teachers’ knowledge and beliefs about psychological problems influence the way they deal with their students’ mental health issues. This study is a preliminary investigation evaluating the effectiveness and feasibility of a School Mental Health Programme (SMHP) developed by the World Health Organization’s Eastern Mediterranean Regional Office (WHO-EMRO) in improving mental health literacy and self-efficacy among school teachers in an inner-city area of urban Lahore.

**Methods:**

Teachers were randomly assigned to 3 days standardized WHO-EMRO School Mental Health Manual based Intervention (n = 118) or to a wait list delayed intervention control group (n = 113). Teachers were assessed pre and post training and at 3 months follow up using measures for mental health literacy (Primary outcome) and self-efficacy. School Heads completed the WHO School Psychosocial Profile and students reported socioemotional skills and psychological problems using Strengths and Difficulties questionnaire at baseline and 3 months post intervention.

**Results:**

Compared with waitlist group, teachers in intervention group presented a significant increase in mental health literacy (*F*_2,181_ = 8.92; *P* < 0.001), as well as better teacher’s self-efficacy in classroom management and student engagement (*F*_2,181_ = *16.45; P* ≤ *0.000* and *F*_2,181_ = *4.65; P* ≤ *0.011*, respectively). Increase confidence in helping students with mental health problems was also noted in the intervention arm (*F*_2,181_ = *15.96 P* ≤ *0.000*). Improvement in overall school environment was also found. No statistical difference in the emotional and behavioural difficulties in students was noticed at 3 months.

**Conclusion:**

This study is one of the first preliminary investigation of WHO-EMRO school mental health intervention in Pakistan. The study showed that intervention led to significant improvement in mental health literacy and self-efficacy among teachers, which was largely sustained over time. Despite a major limitation of lack of clustering and likely contamination affecting follow up outcomes, the study showed promising results in the context of mental health promotion, prevention and early intervention in schools in Lahore, Pakistan. A larger cluster randomised trial is justified, given the level of participant engagement and acceptability by schools.

*Trail Registration*: ClinicalTrials.gov registry (NCT02937714) Registered 13th October 2016, https://register.clinicaltrials.gov.

## Introduction

Unmet child mental health needs are associated with health, social and economic costs [1]. Policy makers and implementers in low and middle-income countries (LMIC) are beginning to focus on public health approaches to meet these needs, with an emphasis on prevention and the use of task-shifting approaches and inter-sectoral collaboration to make mental health care more accessible [1–3]. It is increasingly recognized that schools can be key settings for the delivery of early interventions due to several important aspects: a location where children and adolescents regularly congregate; a nurturing environment focused on learning and development, and; staff who are uniquely attuned to working with children and often the first to observe the signs of mental illness [3]. For any school mental health intervention to have an impact on school culture and practice, teachers need to be involved.

The cornerstone of many mental health initiatives in schools is teachers’ mental health literacy. Mental health literacy, i.e., “knowledge and beliefs about mental disorders which aid their recognition, management or prevention,” is foundational for improving access to care and reducing stigma related to mental illness [4, 5]. Significant research has been done in developed countries to improve mental health literacy among teachers [5, 6]. Teachers own understanding and attitudes about mental health, impact the way they react to student’s mental health issues. Various studies of mental health literacy training have shown positive effect on teachers’ capability to recognize students who are facing difficulties related to mental health. Early identification can lead to early referral and thus may decrease the duration of untreated illness [7, 8]. In high-income countries like Canada and Norway, positive outcomes have been observed in children educational and health outcomes following sustained improvement in the mental health literacy of high-school teachers through specially-designed school mental health curricula [7, 8]. Despite the growing empirical evidence of the positive impact of school mental health approaches in Western countries, there is, to our knowledge, limited evidence of the utility or impact of this approach in LMIC. A study in Tanzania showed that training in mental health literacy and its application in classroom settings led to significant improvement in their knowledge regarding mental health and reduction in stigma [9].

### The WHO-EMRO SMH programme

The World Health Organization Eastern Mediterranean regional office (WHO-EMRO) recognized the need for promoting evidence-based school mental health interventions in resource constrained settings and with a view to address the training needs of educators, developed a manual of school mental health [10]. It is mainly envisioned for all stakeholders (including teachers), involved in the academic process. Its objectives include; helping teachers to recognize the significance of mental health in school settings, become aware of phases of child development, providing them information about evidence-based behavior management strategies and focusing on mental health promotion activities by using whole school approach. Topics covered in manual include: (1) Social-emotional childhood development, (2) Mental Health Promoting Schools (Promotion and Prevention), (3) Addressing Student Mental Health Problems in Your Classroom (and when to refer for additional help) and, (4) Case studies to aid understanding of commonly occurring problems. Standardized school mental health manual handouts are also given to the participants to aid their learning. A systematic evaluation of the programme is lacking.

Pakistan, is the most populous of the WHO’s Eastern Mediterranean region’s 22 countries with over two-thirds of the population classified as youth. Previous studies from Pakistan suggested that around 17% of 5–11-year-old children in schools suffer from emotional and behavioral problems [11]. The significantly high proportion of young population in Pakistan, coupled with high rates of children with unmet mental health needs, requires urgent attention. The emphasis on universal education has seen high levels of enrolment to schools in both rural and urban areas which increases the prospect of accessing many young people through a combination of support between health and education sectors. Given the limited evidence base of effectiveness of the WHO-EMRO school mental health programme, a randomized controlled trial (RCT) of effectiveness of teachers’ training intervention based on the WHO-EMRO Manual of School Mental Health was planned in Urban Lahore, Pakistan.

#### Aims and objectives of the study

This RCT sought to demonstrate the effectiveness of a teacher training programme using the WHO-EMRO Manual of School Mental Health in improving teachers’ mental health literacy, self-efficacy and confidence in helping students with mental health difficulties as compared to a waitlist control group. The primary outcome measure for the trial was teachers’ mental health literacy post-intervention. The secondary objective was to determine if mental health training of teachers led to an indirect improvement in a students’ outcome measure (emotional and behavioral difficulties) and the school psychosocial environment at 3-months post-intervention.

## Methods

### Study setting

The study was conducted in Lahore, the capital of Punjab Province of Pakistan in 2019. The study setting was private secondary schools located in urban Lahore catering to the lower-middle socioeconomic class. The selected schools were matched on broad characteristics including class-size (25–30 students:1 teacher ratio), medium of instruction (English), examination system (Board of Secondary and Intermediate Education, Punjab), and recreational facilities such as a sports ground.

The Institutional Review Board of King Edward Medical University approved the study (Ref 299/RC/KEMU). Detailed study methodology of the trial has also been discussed in the protocol paper [12].

### Study design

We employed an individual randomized controlled trial design where teachers were randomly assigned to either receive training in the school mental health programme immediately or being placed on a wait list to receive training once the trial had finished. The trial was registered at the ClinicalTrials.gov registry (NCT02937714).

### Recruitment/participants

The six schools, were approached through a letter to the Heads of schools inviting them to participate. Principal investigator provided an overview of the study as well as description of WHO-EMRO manual including its rationale, format of interventions proposed and anticipated potential benefit to the target population. Following buy-in from the Heads, similar sessions were organized with teachers of participating schools to clarify any aspect of the project. All teachers were reassured by the Heads that participation was entirely voluntary and would not have any bearing on their employment and that all teachers would eventually be offered the training.

Teachers of all grades (1–10) in the six consenting schools were approached and given written information about the project to seek consent and those willing to participate voluntarily were recruited to the study after written informed consent. Exclusion criteria included teachers who were temporary (had less than 3 months left in the school) and teachers who had not been involved in active teaching in last 6 months. Participants were randomly allocated to the intervention group (receiving the school mental health training during the study) or the waitlist control group (no training till after the end of study) using computer-generated random numbers. Although the teachers in the intervention group were requested to limit their contact and communication from the control group teachers during the training but there may be some likelihood of teachers in two arms having opportunity to share knowledge and practice and influence the teacher outcomes at post intervention*.* The intervention delivery team was not involved in the randomization procedure.

We also recruited a sample of students from the participating schools irrespective of them being taught by intervention or control group teachers. Parents of all students aged 11–16 years currently attending the schools were sent an information letter about the study and details of the training to be delivered to teachers and written consent was sought for their children to complete the study questionnaires. Screening for behavioural and emotional difficulties was done only for those children whose parent or legal guardian gave written consent. We also sought the students’ assent prior to administering the Questionnaire. The participants were free to withdraw at any time during the trial.

Information was also provided to school administration, teachers, the students, and their parents to ensure they were aware of the referral process to a child and adolescent mental health service, should they identify a need for further psychological assessment or treatment. Confidentiality was ensured throughout the study.

### Intervention

The core elements of the intervention are described above. The teachers’ training curriculum based on WHO-EMRO School Mental Health Manual is delivered through a workshop delivered on school premises to teachers in the intervention arm. The training emphasized issues highlighted during in-depth interviews by teachers and school administrators and discussions with relevant stakeholders to make it suitable for local context [13]. The workshop was spread over 3 days with daily 6-h sessions delivered face-to-face over a 2-week period. The training consisted of lectures, discussions, and role-plays. Teachers had the option to decline to participate at any stage of the study.

The workshop was conducted by the principal investigator (PI) along with one assistant (psychologist trained by the PI). The PI was trained by one of the National Trainers trained by the WHO EMRO Master Trainer.

### Outcome measures

#### Primary outcome

##### Teachers’ mental health literacy

The primary outcome was teachers’ mental health literacy, immediately post intervention. It was assessed using a questionnaire developed by the WHO to evaluate their trainings of teachers in the Eastern Mediterranean region and adapted for cultural appropriateness by a team of Pakistani mental health experts. The first section of the questionnaire has 30 multiple choice questions: for example, “Frequently leaving the class due to pains and aches that do not appear on weekends or holidays may be a warning sign of mental illness” and; “Students with anxiety problems may freeze or be unable to participate in activities”. Possible responses are ‘true’, ‘false’ and ‘I don’t know’. The second section has six vignettes with 5 questions each of possible interventions to be considered by school teachers. Each question has ‘yes’, ‘no’ and ‘I don’t know’ format. Participants are told to choose only one option per question and were encouraged to mark ‘I don’t know’ rather than guessing if unsure. Each correct answer was scored as 1. The questionnaire generates a maximum score of 60. Cronbach’s alpha for scale was 0.78, indicating a high level of internal consistency for the questionnaire with this specific sample.

### Secondary outcomes

#### Teachers’ sense of self efficacy

Teacher self-efficacy refers to beliefs and confidence teachers have in their ability to successfully implement actions which will positively influence student learning. This was assessed using the Teachers’ Sense of Self Efficacy Scale (TSES) [14]. The questionnaire has 12 statements and each statement is rated on 9-point Likert scale. For this study, the teachers’ efficacy in ‘student engagement’ and ‘classroom management’ subscales were used, as they were related well to the content of the intervention. Responses of items of each subscale are added to generate a total subscale score (0–36). Cronbach’s alpha for teachers’ efficacy in student engagement was 0.9 and for classroom management subscale was 0.88, indicating a high level of internal consistency for the scale with this specific sample.

#### Confidence in providing help

Teachers were asked “How confident do you feel in helping a student with a mental health problem?” on a Likert scale with options (Not at all, A little bit, Moderately, Quite a bit, Extremely) [15].

#### Feedback about the training

A feedback questionnaire was also used to obtain intervention group teachers views regarding the content, delivery and quality of training at the end of training with view to be used to further adapt and refine the training & manual.

#### The strengths and difficulties questionnaire (SDQ)

Data was collected from all eligible students at baseline and 3 months following the intervention using the Strengths and difficulties Questionnaire (SDQ), a universally validated tool to screen for behavioural and emotional problems. The scale has been translated and validated in Urdu language in Pakistan [16, 17]. We used the self- reported SDQ (suitable for 11–16-year-olds). It measures 25 attributes, with subscales generating scores for conduct, hyperactivity, emotional, peer problems and prosocial behaviour. Statements are rated on a three-point Likert scale:0 (not true), 1 (somewhat true) and 2 (certainly true). All scales excluding the last are added to generate a total difficulties score (0–40). Total difficulties and subscale scores are coded in normal, borderline and abnormal categories.

#### School psychosocial profile questionnaire

Data was collected from all participating schools using the WHO Psychosocial Profile Questionnaire [18]. This questionnaire groups school characteristics in seven quality areas. Questions in each area are scored from 1 (not at all) to 4 (very much). The average score of each quality area is calculated. Data was collected from Head Teachers at baseline and 3 months following the intervention.

#### Data collection and management

Data was collected from both intervention and control group teachers at baseline, immediately after the training workshop, and 3 months following the workshop. All teachers’ measures were self-administered using anonymized questionnaires. Blinding of participants was not possible as post-test and follow-up questionnaires were self-completed by teachers. The students were also assessed at baseline and 3 months after the intervention. The anonymized questionnaires were self-administered in the classroom setting. Research staff facilitated data collection from teachers and students, explaining the procedures and transporting the data confidentially for input. All data were stored in locked premises. All staff conducting data entry and analysis were blind to the intervention status.

### Sample size calculations and statistical analysis

Sample size of 220 teachers (110 teachers in intervention and control group teachers each) was estimated using 90% confidence interval, 5% absolute precision with expected improvement in mental health literacy of 10% in the intervention group compared to 1% in the wait-list control [15].

The data were coded, entered and analyzed using the SPSS 20 statistical package A description of individuals who participated in the two study arms were compiled using descriptive statistics such as mean and standard deviation, frequency and percentages. Aggregates of the mean item score were created for each scale at three timelines (baseline T0, immediately following intervention T1, and 3 months following intervention T2). A repeated measures analysis of variance (ANOVA) was used to evaluate primary outcome (mental health literacy), considering 2 conditions (control and intervention) and 3 distinct periods [baseline (T0), postintervention (T1), and follow up(T2)]. The time × group interaction effects were assessed to investigate differences regarding the magnitude of change on dependent variables (teachers’ mental health literacy, teacher’s self-efficacy in classroom management and student engagement and confidence in helping students with mental health problems) between the experimental and control groups. Student outcome measures for schools and psychosocial environment was compared at baseline and 3 months follow-up using the t test. Associations were considered significant at the 5% level. Data on screening, refusals and dropouts were reported as per Consolidated Standards of Reporting trials (CONSORT) guidelines for participant flow through the trial [19].

## Results

### Participants and attrition

**Fig. 1:**
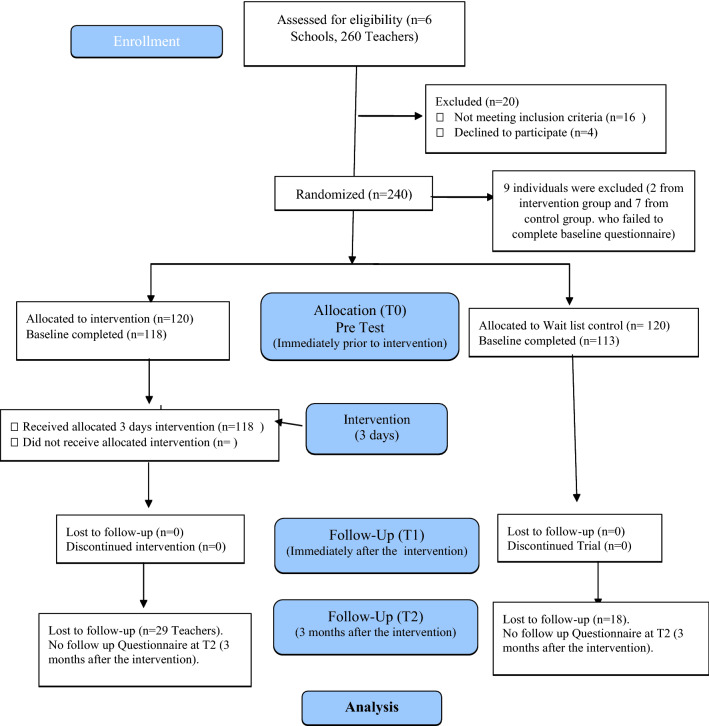
Flow diagram of the randomized controlled trial

The recruitment and flow of participants through the trial is presented in Fig. 1. Six large representative schools were selected and agreed to participate in the trial. All teachers in these schools (n = 260) were approached to take part in the study. Two hundred and fifty-six teachers gave consent to be randomized, 16 teachers were ineligible as they did not meet inclusion criteria. After individual randomization of 240 teachers, 7 teachers (2 from intervention group and 7 from control group) were dropped as they failed to complete the baseline questionnaire. Thus 231 teachers were randomized to either intervention (118) or wait list control (113) groups. All participants who completed the baseline questionnaire were included in the analysis.

All participants in both groups completed the follow-up assessments at Time point 1 (T1) (Post intervention). Twenty-nine participants in the intervention arm and 18 in the control arm were lost at the three-month follow-up. Thus, at 3 months, the trial had a 75.4% (89/118) response rate in the intervention arm and 84% (95/113) in the control arm. There was no difference in the characteristics of participants who dropped out in both groups.

### Participants’ characteristics

#### Teachers

Table 1 compares the socio-demographic characteristics of the teachers in intervention and control arms at baseline. Factors pertaining to age, gender or classes being taught were evenly distributed between the intervention and control arm. No significant differences were observed at baseline between the two groups on mental health literacy and perceived teacher’s self-efficacy. (P value > 0.05).

**Table 1:** Baseline characteristics of teachers in intervention and control groups

Characteristics	Intervention group N (%)	Control group N (%)
Teachers (n)	118	113
Age: mean (SD)	32.6 (9.0)	32.3 (10.3)
Gender		
Male	19 (16.1)	16 (14.2)
Female	99 (83.9)	97 (85.8)
Education		
Matric	2 (1.7)	7 (6.2)
Intermediate	12 (10.2)	16 (14.2)
Bachelors	17 (14.4)	24 (21.2)
Masters	73 (61.9)	64 (56.1)
Higher qualification	14 (11.9)	2 (1.8)
Classes being taught		
Primary	39 (33.1)	48 (42,5)
Secondary	47 (39.8)	37 (32.7)
Both	32 (27.1)	28 (24.8)
Teaching experience in years. Mean (SD)	7.6 (6.0)	9.52 (7.6)
Baseline Mental Health Literacy Questionnaire score T0 (from a maximum score of 60). Mean (SD)	37.39 (5.80)	37.61 (6.82)
Baseline Teacher’s Self Efficacy in classroom Management T0 (from a maximum score of 36). Mean (SD)	28.26 (5.71)	29.67 (5.33)
Baseline Teacher’s Self Efficacy in student engagement T0 (from a maximum score of 36). Mean (SD)	28.79 (5.65)	29.38 (5.24)
Confidence in helping students with mental health problems Baseline.T0 (from a maximum score of 5). Mean (SD)	3.14 (0.96)	3.54 (0.64)

#### Students

Out of a total of 1080 students (aged 11–17 years,) 836 (77.4%) provided parental consent for their participation in the study. 597 (71.4%) were females and 239 (28.6%) were male students.

### Teacher outcomes

Table 2 shows descriptive data related to repeated measures analysis of variance. Analysis of Mental health literacy scores revealed a significant time effect (*F*_2,181_ = *41.5; P* ≤ *0.000*; $\eta_{p}^{2}$  = 0.314). Furthermore time × group interaction (*F*_2,181_ = *38.66; P* ≤ *0.000*; $\eta_{p}^{2}$ = 0.314) indicated that the intervention group had significant increase in mental health literacy compared with the control group.

**Table 2:** Comparison of teacher outcome variables between the intervention and control arms at baseline, post intervention (T1) and follow up (T2)

	Intervention	Control	Group difference	Time effect		Group effect		Time * group effect	
	Mean (SD)	Mean (SD)	Mean^a^ (95% CI)	Sig	$\eta_{p}^{2}$	Sig	$\eta_{p}^{2}$	Sig	$\eta_{p}^{2}$
Mental Health Literacy									
Baseline (T0)	37.60 (5.39)	36.86 (6.71)	5.70* (4.26–7.14)	*F* = *41.51* *P* ≤ 0.000**	0.314	*F* = *61.36* *P* ≤ 0.000**	0.252	*F* = *38.66* *P* ≤ 0.000**	0.314
Post intervention. (T1)	46.30 (6.05)	36.12 (6.78)							
Follow-up (3 months). (T2)	42.84 (6.05)	35.94 (6.70)							
Teacher’s Self-efficacy in classroom management									
Baseline (T0)	28.01 (6.10)	29.67 (5.68)	0.631 (− 0.628 to 1.89)	*F* = *12.49* *P* ≤ 0.000**	0.121	*F* = *0.977* *P* = *0.324*	0.005	*F* = *16.45* *P* ≤ 0.000**	0.154
Post intervention. (T1)	31.46 (3.73)	29.17 (5.03)							
Follow-up (3 months). (T2)	30.16 (4.38)	28.89 (5.31)							
Teacher’s Self-efficacy in student engagement									
Baseline (T0)	28.53 (5.99)	29.31 (5.58)	0.305 (− 0.937 to 1.54)	*F* = *2.89* *P* ≤ 0.05*	0.031	*F* = *0.235* *P* = *0.629*	0.001	*F* = *4.65* *P* ≤ 0.011*	0.049
Post intervention. (T1)	29.94 (4.97)	28.43 (4.93)							
Follow-up (3 months). (T2)	28.51 (5.43)	28.33 (5.00)							
Confidence in helping students with mental health problems									
Baseline (T0)	3.08 (0.650)	3.52 (0.650)	0.102 (− 0.065 to 0.269)	*F* = *8.38* *P* ≤ 0.000**	0.085	*F* = *1.46* *P* = *0.228*	0.008	*F* = *15.96* *P* ≤ 0.000**	0.150
Post intervention. (T1)	3.80 (0.786)	3.40 (0.735)							
Follow-up (3 months). (T2)	3.65 (0.893)	3.31 (0.839)							

Data are presented as means (standard deviation) for all outcome measures

*CI* confidence interva, $\eta_{p}^{2}$ effect size partial eta-squared

^a^Mean Difference (Control-Intervention); adjustment for multiple comparisons (Bonferroni)

.*The mean difference is significant at the 0.05 level

**P value < 0.001

For teacher’s self-efficacy in classroom management and student engagement, there was a significant time × group interaction (*F*_2,181_ = *16.45; P* ≤ *0.000* and *F*_2,181_ = *4.65; P* ≤ *0.011*, respectively), showing a greater benefit for individuals in the intervention group.

A time × group interaction was also observed (*F*_2,181_ = *15.96 P* ≤ *0.000*)., with participants in the intervention condition showing a greater increase in confidence in helping students with mental health problems than participants in the control group. (Table 2).

### Secondary outcome measures

#### School environment

There were 6 participating schools in the trial. Table 3 compares the various domains of The Psycho-Social Profile Questionnaire (PSE) at baseline and 3 months follow-up. The mean scores in some domains (providing a friendly; rewarding and supportive atmosphere; supporting cooperative and active learning; valuing the development of creative activities; connecting school and home life through involving parents) were improved significantly while no significant improvement was seen in domains of forbidding physical punishment and violence; not tolerating bullying, harassment and discrimination, and; promoting equal opportunities and participation in decision making.

**Table 3:** Comparison of The Psycho-Social Profile Questionnaire (PSE) at baseline and 3 months after teachers training

		T0 (baseline)	T1 (3 months post intervention)	***t***	95% CI		*p*
		Mean (SD)	Mean (SD)		LL	UL	
The psycho-Social Profile Questionnaire							
Domain 1	Providing a friendly, rewarding and supportive atmosphere	66.67 (2.06)	76.50 (3.27)	− 13.98	− 11.64	− 8.02	0.000**
Domain 2	Supporting cooperative and active learning	25.17 (2.48)	26.50 (1.87)	− 13.44	− 6.75	− 4.58	0.001*
Domain 3	Forbidding physical punishment and violence	55.83 (3.12)	56.33 (3.01)	− 2.23	− 1.07	0.075	0.076
Domain 4	Not tolerating Bullying, harassment and discrimination	48.83 (5.41)	49.33 (5.12)	− 2.23	− 1.07	0.075	0.076
Domain 5	Valuing the development of creative activities	26.00 (1.09)	30.33 (1.96)	− 5.70	− 6.28	− 2.37	0.002**
Domain 6	Connecting school and home life through involving parents	37.67 (3.14)	42.83 (3.18)	− 9.52	− 6.56	− 3.77	0.000**
Domain 7	Promoting equal opportunities and participation in decision making	36.50 (1.04)	36.83 (1.47)	− 1.58	− 0.87	0.20	0.175

Data are Mean (SD)

Paired T test applied

*P value < 0.05, **P value < 0.001

#### Emotional and behavioural difficulties in students

Emotional & behavioral difficulties in students [ages 11–17) in participating schools were assessed through the Strengths and Difficulties Questionnaire. Table 4 compares the SDQ scores at baseline and 3 months follow-up. The mean scores in the different subscales of SDQ was less than that at the baseline, indicating that there was a slight improvement in the students emotional and behavioral difficulties, but this was not statistically significant (P value > 0.05).

**Table 4:** Comparison of Students emotional and Béhavioral Difficulties (SDQ scores) for students at baseline and 3 months after teachers training

Strengths and difficulties questionnaire	T0 (baseline)	T1 (3 months post intervention)	t	95% CI		P value
	Mean (SD)	Mean (SD)		LL	UL	
SDQ (total)	13.30 (5.14)	12.73 (4.75)	1.61	− 0.12	1.25	0.108
SD (conduct)	3.10 (1.73)	3.01 (2.16)	0.56	− 0.22	0.39	0.575
SDQ (hyperactivity)	3.14 (1.82)	3.05 (1.75)	0.64	− 0.19	0.37	0.522
SDQ (emotional)	3.69 (2.33)	3.50 (2.18)	1.28	0.09	0.47	0.199
SDQ (peer problems)	3.34 (1.66)	3.31 (1.78)	0.19	− 0.25	0.30	0.848
SDQ (prosocial)	7.90 (1.73)	8.00 (2.07)	0.88	− 0.17	0.47	0.376

Paired T test applied

Data are Mean (SD)

#### Acceptability of the intervention

Feedback was obtained from teachers on the acceptability of the intervention, using a structured feedback questionnaire. A total of 96 respondents [90 teachers among 118 in intervention group (76%) and all 6 headteachers(100%)] returned the feedback questionnaire. All Head Teachers (6, 100%) were cooperative and felt school mental health training was important. However, there were logistical challenges in arranging cover for classes of teachers in the intervention group. The teachers (85, 94%) recommended future training to be held either during school vacations or integrated into ongoing professional development activities. The venue for trainings and number of participants in each training sessions were deemed satisfactory. Most of the teachers (86,95%) who gave feedback were positive about the training and felt more confident in recognizing and addressing mental health problems in students as well as knowing when to refer a student for additional help. Seventy teachers (77%) felt that modules on detection and management of learning difficulties, Epilepsy and psychosomatic problems needed to be expanded as these being very common presentations. All teachers (90,100%) expressed the need for teacher stress management to be incorporated in training. They felt that teachers’ own stress often made it difficult for them to adequately help students or deal with their parents.

## Discussion

This study examined the impact of teachers’ training on their mental health literacy, self-efficacy, and confidence in dealing with common mental health problems presenting in their students. The intervention produced a positive impact on all these three domains. Although there was improvement in the school psychosocial environment and student socioemotional health, but lack of clustering means that secondary outcomes are likely to have been affected by contamination. The short time frame and small sample size did not allow us to detect any significant changes on students’ socioemotional health. However, as teachers are the key mediators of the intervention, our findings are important as they demonstrate a strong positive effect of the teachers’ training on these intermediate variables on the pathway to improvement of the mental health of children. A cluster RCT would have been ideal, but the lack of resources did not allowed it. Despite major limitation of lack of clustering possible contamination, study had many strengths including successful training of teachers, the feasibility of integrating the intervention, and good response rates. The results indicates that the intervention merits larger scale evaluation in a cluster randomised controlled trial.

Mental health problems in children carries a high cost. The evidence that significant burden of mental illnesses originate in young age and the key to prevention and recovery are early interventions, are leading to recommendation for higher investment in child mental health. Promoting mental health of young population through schools is a cost-effective investment that can improve both health and education simultaneously, and assist in the realization of the United Nations’ sustainable development goals (SDGs) 3 and 4. Other studies have found that involvement with and training of teachers in issues relating to child mental health, as in the intervention tested here, leads to improved access to care, and form an important task-sharing and collaborative care strategy in LMIC with limited resources [20]. Evidence from LMIC including Asian countries is scarcer. In Pakistan, a teachers’ training program focused on ADHD led to an improvement in both general awareness and knowledge of ADHD in the teachers [21] but outcomes in the students were not measured. A more comprehensive rural school mental health program focusing on mental health literacy of students as well as teachers through a number of classroom and school-based activities embedded in the school routine for 4–6 months in Rural Rawalpindi led to major improvements in the mental health awareness of the school children [22]. The current study extends such programmes by including guidelines through which teachers took practical steps to manage common problems in the school settings successfully, leading to strong trends of improved mental health in the children. However, we observed that positive effect of improvement in mental health literacy in our sample decreased slightly over time progressed, and this highlights the need for periodic booster training sessions for teachers to maintain the effects of the program.

Our study also found a positive improvement in teachers’ self-efficacy. This construct reflects a personal belief in one’s own ability to satisfactorily execute the various tasks associated with and required by their job, in addition to create an adequate learning environment [23]. Feeling better equipped with training, teachers felt able to effectively manage their classes alongside developing supportive and positive relationship with students. Like other teachers training programmes, the intervention emphasizes the importance of the relationship between adults and children, and the awareness of factors that can reinforce negative behaviors in class. Teachers have a much broader role in development of children rather than intervening only when a mental health issue leads to learning impairment. Thus, better classroom management and engagement with students helps in promoting positive mental health and leading to better outcomes. Conversely, lack of confidence in helping students with mental health issues due to lack of knowledge among teachers is a barrier to meeting their educational objectives. Our study adds to this evidence, showing that improving mental health literacy leads to better self-efficacy as well as increase confidence in ability to support vulnerable students in school setting.

The study was not designed specifically to assess improvement in students emotional and behavioural difficulties; however, it was envisaged that better mental health literacy in teachers would lead to improvement in students’ mental health. We found an overall reduction in the mean scores of students in the different subscales of Strengths and Difficulties Questionnaire, but this did not achieve statistical significance. Our study was not powered to detect changes in students’ outcomes. Furthermore, only half the teachers were trained while the other half were wait-list controls and this may have diluted the effect of the intervention. As mentioned earlier, likely contamination also affects all of the follow-up and secondary measures. Finally, due to time and resource limitations, we were able to follow up the students 3 months after the teachers’ trainings and this time period is likely to be too short to achieve the desired outcomes in students. Also However, trends towards improvement in all domains of the SDQ are promising. Well-powered cluster randomized trials are required to evaluate the short- and long-term impact on students’ mental health. Studies from high-income countries found mixed results in students where their teachers had undergone mental health literacy trainings. In urban Colorado, 43.7% of students, a significantly higher proportion than controls showed significant improvement in their functioning following a school-based intervention [24]. It is likely that school based mental health programmes shift the paradigm from reactive and individual to preventive and universal and likely to benefit everyone by reduction in mental health problems faced amongst students as well as functional impairment associated with it.

The school environment exerts an important role in student development and health including mental health. Our results showed improvement in many aspects of the school environment, however lack of clustering and possible risk of contamination (when there is a mix of teachers from both trial arms at the same school at 3 months follow up) the improvement cannot be attributed as a result of the intervention alone. This supports recommendations that the priority of school mental health initiatives should be to improve the environment in schools and overall health rather than treatment of psychopathology [25]. School environment is considered very relevant to school based anti-stigma campaigns and help seeking attitudes. We did not observe any significant improvement in some aspects of school environment which shows that these aspects of the intervention may need to be reinforced.

The study has important implications for the education and health sectors, as well as policy and research. Teachers training in school mental health should be considered an important component of pre-service or in-service training of teachers. School mental health services need to be coupled with better quality mental health services in the primary health care for referral of cases from the schools, along with referral linkages with higher care levels for management of cases needing specialized care. Positive trends from the current study support the development of further research on a larger scale, which is urgently needed. The study also highlighted the need for further refinement in WHO intervention manual. There need to be focus on teachers own mental health issues and stress management techniques. Psychosomatic and developmental conditions need to be addressed. Possibility of booster sessions for teachers to ensure sustained improvement in knowledge and attitudes related to mental health issues should also be considered.

### Limitations and strengths of the study

Despite the indication that the WHO school mental health intervention was effective in the current study for improving teacher’s mental health literacy and self-efficacy, we recognize that the study has some limitations. A major methodological concern is that individual randomization could lead to the risk of contamination thus conclusions that have been drawn need to be considered with this limitation in mind This would increase the risk of beta error, and future studies should use cluster randomized designs, especially if the primary outcomes relate to child mental health. The study was based on representative sample of teachers from private schools in inner city areas, thus it is not possible to generalize the results to teachers in public sector schools. Another limitation is that the sample was predominantly composed of female teachers and therefore we cannot tell if male teachers respond differently to training. The attrition rate between baseline and 3-months follow-up was relatively high although all participants attended the whole training and completed the assessments at post-intervention. It is likely that the attrition rate was due to rapid turnover of teachers in these small unregulated private schools or competing pressures of work making it a low-priority activity for some teachers. Another limitation is the relatively short duration of the follow-up assessments which limit our understanding of the long-term impact of the intervention, especially in child outcomes. For these outcomes, we used a pre-post design, which is considered relatively weak. The study evaluated the improvement in mental health literacy and other variables, but it was beyond the scope of the study to assess the impact on participants’ application of the new knowledge and skills in actual classroom settings. This should be considered in future research in this area.

## Conclusion

There is a high burden of mental disorder among children and adolescents in LMICs. Engaging teachers through task-sharing approaches can help in reducing the treatment gap for these disorders. The WHO-EMRO School mental health manual-based intervention was effective in improving teachers’ knowledge and self-efficacy as well as confidence in ability to help students facing mental health difficulties. Notably,here were possible additional benefits of improved school environment and a positive trend towards improvement in students’ behavioural and emotional difficulties. Despite the limitations of the study, a larger cluster randomised trial is justified, given the level of participant engagement and acceptability by schools. The study shows that education and health sector can work in close collaboration and make a joint policy to promote school mental health. The intervention holds potential for widespread applicability in Pakistan and elsewhere in the region and merits further exploration and evaluation.

## Data Availability

All data generated or analysed during this study are included in this published article [and its Additional files].

## Abbreviations

SMHP: School Mental Health Programme
WHO-EMRO: World Health Organization’s Eastern Mediterranean Regional Office
LMIC: Low and middle-income countries
PI: Principal investigator
TSES: Teachers’ Sense of Self Efficacy Scale
SDQ: Strengths and difficulties questionnaire
ADHD: Attention deficit hyperactivity disorder
